# Effects of Resveratrol on Methotrexate-Induced Testicular Damage in Rats

**DOI:** 10.1155/2013/489659

**Published:** 2013-07-28

**Authors:** Esin Yuluğ, Sibel Türedi, Ahmet Alver, Süleyman Türedi, Cemil Kahraman

**Affiliations:** ^1^Department of Histology and Embryology, Faculty of Medicine, Karadeniz Technical University, 61080 Trabzon, Turkey; ^2^Department of Medical Biochemistry, Faculty of Medicine, Karadeniz Technical University, 61080 Trabzon, Turkey; ^3^Department of Emergency Medicine, Faculty of Medicine, Karadeniz Technical University, 61080 Trabzon, Turkey

## Abstract

This study investigated the probable protective effects of resveratrol (RES), an antioxidant, against methotrexate- (MTX-) induced testis damage. Twenty-four male Sprague Dawley rats were randomly divided into four groups: control, RES, MTX, and MTX + RES groups. Rats were sacrificed at the end of the experiment. Plasma and tissue malondialdehyde (MDA) levels, superoxide dismutase (SOD) and catalase (CAT) activity in tissue, testicular histopathological damage scores, and testicular and epididymal epithelial apoptotic index (AI) were evaluated. The MTX group had significantly higher plasma and tissue MDA levels and significantly lower SOD and CAT activity than those of the control group. In the MTX + RES group, plasma and tissue MDA levels decreased significantly and SOD activity rose significantly compared to the MTX group. The MTX group had significantly lower Johnsen's testicular biopsy score (JTBS) values than those of the control group. JTBS was significantly higher in the MTX + RES group than in the MTX group. AI increased in the testis and epididymis in the MTX group and significantly decreased in the MTX + RES group. Our results indicate that RES has protective effects against MTX-induced testis damage at the biochemical, histopathological, and apoptotic levels.

## 1. Introduction

The use of chemotherapeutics is known to cause acute toxic effects in multiorgan systems [1]. Permanent azoospermia and infertility have been reported as side effects of chemotherapeutic drugs in males [2]. Methotrexate (MTX) is a folic acid antagonist agent used for chemotherapeutic purposes in malign tumors (acute lymphoblastic leukemia, non-Hodgkin's and lymphoma, breast cancer, malignancies of the head and neck, among others) and nonneoplastic diseases (particularly rheumatoid arthritis) [3]. Previous studies have reported damage (disorganization and vacuolization) in the seminiferous tubules of the testis, a decrease in sperm numbers, and sperm DNA damage following administration of MTX [4, 5]. Oxidative stress has been reported to play an important role in the pathogenesis of MTX-induced testicular damage [6]. Atrophy in the testicular seminiferous tubules and apoptosis in spermatocytes have been linked to an increase in reactive oxygen radicals (ROS) [3, 5]. Studies have recently investigated the use of antioxidant materials in order to reduce the side effects resulting from MTX administration [5, 7]. 

Resveratrol (trans-3,5,4′-trihidroksi-stilben) (RES) is a phytoalexin with antioxidant properties and is found in a wide range of foods, especially grapes. In vivo and in vitro studies in recent years have shown that RES particularly protects spermatocytes against lipid peroxidation and increases testicular sperm numbers and sperm motility [8]. It has also been shown to increase sperm production [9], reduce apoptosis in germinal cells [10], and protect against environmental toxins [11]. Previous studies have reported that RES is a potent antioxidant and that it exhibits this activity by increasing antioxidant enzyme release and reducing lipid peroxidation [12]. 

The purpose of this study was to investigate the role of oxidative stress in MTX-associated testicular damage and also to show the probable protective effects of RES against MTX-induced testicular damage at the histopathological and biochemical levels.

## 2. Methods

### 2.1. Animals

This was a randomized, controlled animal study. All animals were kept at room temperature, 22 ± 2°C, in a controlled 12/12 h light/dark cycle. Standard laboratory chow and water were given. All animals received human care according to the criteria outlined in the “Guide for the Care and Use of Laboratory Animals” published by the National Institutes of Health. The study was approved by the Karadeniz Technical University Animal Care and Ethical Committee.

### 2.2. Experimental Protocol

Twenty-four adult male Sprague Dawley rats (8 weeks old) were used. The rats were divided at random into 4 groups of 6 animals each. No procedure was performed in the control group. The RES group was administered 20 mg/kg resveratrol (R5010-500 mg, Sigma-Aldrich, St. Louis, MO, USA) intraperitoneally (i.p.) for 10 days. The MTX group was given 30 mg/kg methotrexate (Koçak Farma, Tekirdağ, Turkey) on day 7 of the experiment. The MTX + RES group was given 20 mg/kg RES i.p. for 7 days, on the 7th day, MTX 30 mg/kg was administered i.p., and RES continued to be given for a further 3 days. At the end of the 10 days, laparotomy was performed on all rats under anesthesia. The abdominal cavity was quickly opened and the bilateral testis and epididymis extracted. All animals were sacrificed by exsanguination at the end of the procedure.

### 2.3. Biochemical Analysis

Plasma malondialdehyde (MDA) levels were determined using the method described by Yagi [13]. Briefly, to 0.3 mL of serum was mixed 2.4 mL of 0.08 N H_2_SO_4_ and 0.3 mL of 10% phosphotungstic acid. After being allowed to stand at room temperature for 5 min, the mixture was centrifuged at 1600 g for 10 min. Discard supernatant and sediment was suspended in 4 mL of distilled water. Subsequently, 1 mL of 0.67% thiobarbituric acid was added, and the mixture was heated in boiling water for 60 min. The formed colour was extracted into n-butanol. The mixture was centrifuged at 1600 g for 10 min. The absorbance of the organic layer was read at 532 nm. Tetramethoxypropane was used as a standard and MDA levels were calculated as nmol/L.

MDA levels in testicular samples were measured by the method of Uchiyama and Mihara [14]. Briefly, a piece of testicular tissue was minced and homogenized in an ice-cold 1.15% KCl solutioncontaining 0.50 mL/L Triton X-100 using an Ultra-Turrax T25 homogenizer. The homogenate (0.5 mL) was mixed with 3 mL of 1% H_3_PO_4_. After adding 1 mL of 0.67% thiobarbituric acid, the mixture was heated in boiling water for 45 min. The color phase was extracted into n-butanol. The mixture was centrifuged and absorbance of the organic layer was read at 532 nm. Tetramethoxypropane was used as a standard, and MDA levels were calculated as nanomoles per gram wet tissue.

Superoxide dismutase (SOD) and catalase (CAT) activities were determined in the remaining part of the testicular tissue. The sample was homogenized in an ice-cold Tris-HCL buffer (50 mM, pH 7.4) containing 0.50 mL/L Triton X-100. SOD activities were measured by reduction of nitroblue tetrazolium by xanthine-xanthine oxidase system [15]. Formazon formation was assessed spectrophotometrically at 560 nm. Enzyme activity leading to 50% inhibition was taken as one unit and bovine erythrocytes SOD were used as standard. Results are expressed as U/g tissue protein. CAT activity was determined by the method of Aebi [16]. This method is based on the principle that the absorbance at 240 nm decreases because of dismutation of H_2_O_2_ and results are expressed as k/g tissue protein (k, the first order kinetic constant (sec^−1^) for disappearance of H_2_O_2_). Protein concentrations were determined according to Lowry's method [17].

### 2.4. Histopathological Staining and Analysis

The right testis and epididymis tissues were fixed, dehydrated, and embedded in paraffin. Tissues were then stained with haematoxylin and eosin (H&E). All testicular histology was assessed blind by a histologist. Light microscopy (Olympus BX-51; Olympus, Tokyo, Japan) was used for the evaluations. Testis sections from each study group were evaluated for structural changes. Johnsen's tubular biopsy score (JTBS) was used for the semiquantitative evaluation of spermatogenesis in 20 seminiferous tubules from each testicular section [18]. In each group, testicular tubule sections were classified by degrees, ranging from 1 to 10. In this classification, 10, expressed complete spermatogenesis and regular structure; 9, many spermatozoa present and disorganized tubules; 8, only a few spermatozoa present; 7, no spermatozoa but many spermatids present; 6, no spermatozoa, only a few spermatids present; 5, no spermatozoa or spermatids but many spermatocytes present; 4, few spermatocytes present; 3, only spermatogonia present; 2, no germ cells, only Sertoli cells present; and 1, complete absence of germ cells and spermatogenesis. JTBS was calculated by dividing the sum of all scores by the total number of seminiferous tubules examined.

### 2.5. TUNEL Analysis

The TUNEL (terminal deoxynucleotidyl transferase (TdT) deoxyuridine triphosphate nick end labeling assay) technique was used to determine apoptosis in the testis and epididymis. TUNEL analysis was performed using an in situ cell death detection kit, POD, (ROCHE, Mannheim, Germany) in line with the manufacturer's instructions. Color was then developed with a 3,3′-diaminobenzidine including kit (DAB, Sigma, St. Louis, MO, USA). DNA fragmentations were observed in seminiferous tubule germinal cells and epithelial cells of the epididymal canal. Brown-stained TUNEL (+) cells were regarded as apoptotic. TUNEL (+) cell numbers in 20 seminiferous tubule and 20 epididymal canal sections in each testis were evaluated at ×400 magnification using the Analysis 5 Research program (Olympus Soft Imaging Solutions, Münster, Germany). The proportion of TUNEL (+) apoptotic cells in the testis and epididymis to total cells was taken as the testis apoptotic index (TAI) [19] and epididymal apoptotic index (EAI).

### 2.6. Statistical Analysis

Kruskal-Wallis analysis of variance was used to compare differences between group parameters. Dual comparisons between groups exhibiting significant values were evaluated using the Bonferroni corrected Mann-Whitney *U* test. Statistical significance for all tests was set at *P* < 0.05. All results were expressed as means (±) standard deviation (SD).

## 3. Results

All rats survived without major complications throughout the experiment. 

### 3.1. Biochemical Results

Biochemical results for the experimental groups are given in Table 1. Tissue and plasma MDA concentrations in the MTX group were significantly higher compared to the control and RES groups, while SOD and CAT activity were significantly lower in testicular tissue. Tissue and plasma MDA concentrations in the MTX + RES group were significantly lower compared to the MTX group, while there was a significant rise in SOD enzyme activity.

### 3.2. Light Microscopic Evaluation

**Figure 1:**
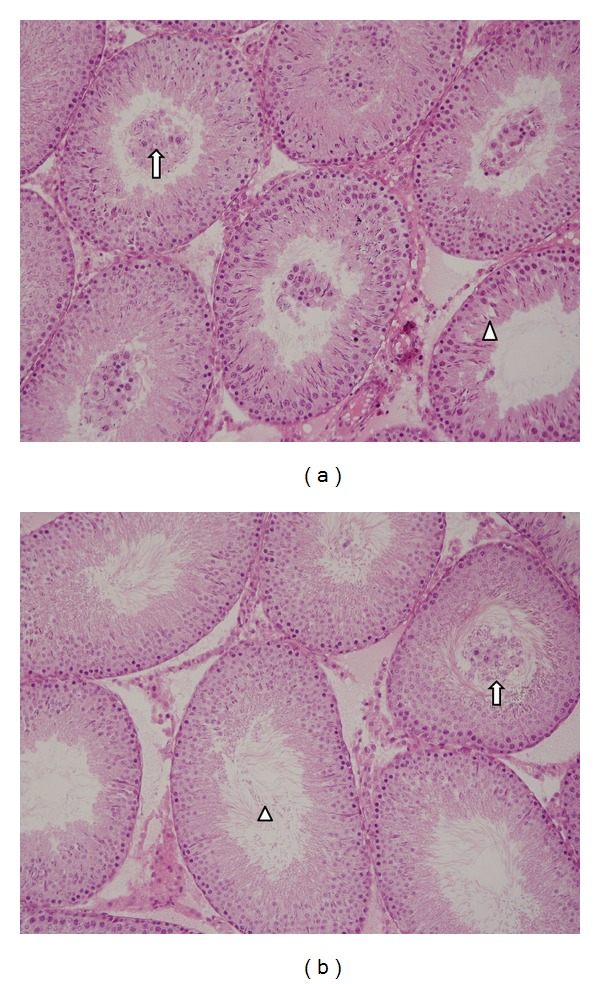
(a) In the MTX group, occasional disorganization in the seminiferous tubule epithelium (arrow head) and immature germinal cells in the seminiferous tubule lumen (arrow). (b) In the MTX + RES group, very few immature germinal cells (arrow) and a very large number of spermatozoa (arrow head) in the seminiferous tubule lumen (H&E ×200).

Histopathological results for the groups are given in Table 2. Normal testis seminiferous tubule morphology was observed in the control and RES groups. In the MTX group, widespread loss of spermatozoa in the seminiferous tubular lumens and accumulation of immature germinal cells in the lumen were observed (Figure 1(a)). Testicular architecture was close to normal morphology in the MTX + RES group. Rare germinal epithelial cells were encountered in the seminiferous tubule lumen (Figure 1(b)). At analysis of spermatogenesis in the testis, the MTX group had significantly lower JTBS compared to the control group, and the MTX + RES group had higher JTBS values compared to the MTX group. 

**Figure 2:**
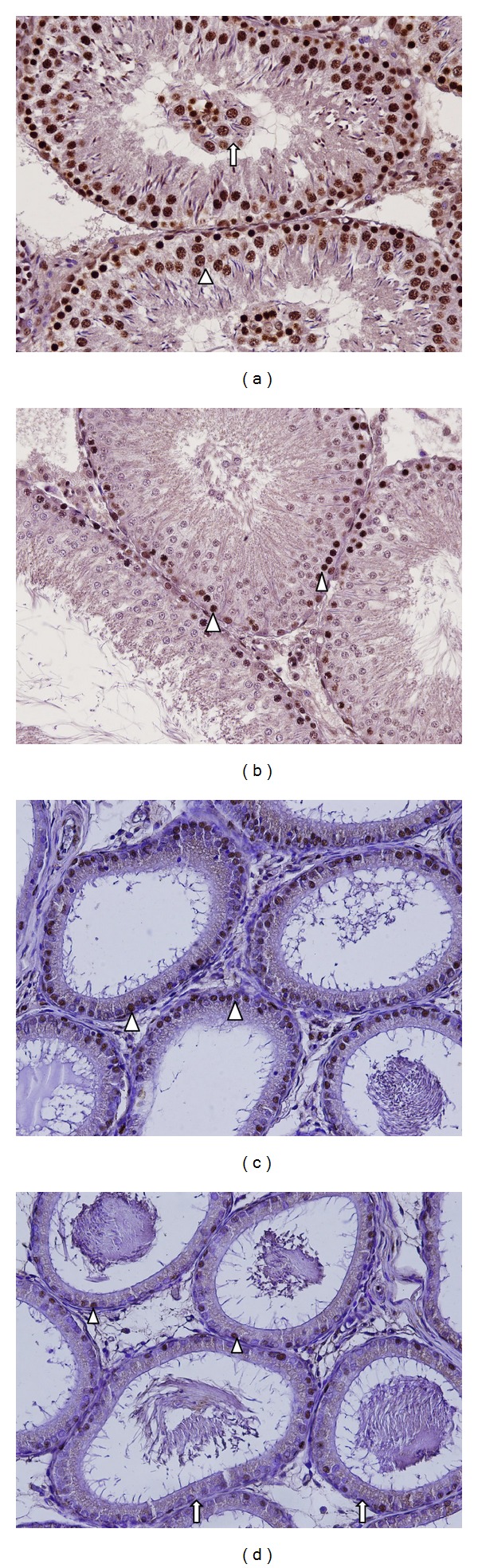
(a) In the MTX group, widespread apoptotic germinal cells in seminiferous tubule germinal epithelium (arrow head) and in the lumen (arrow). (b) In the MTX + RES group, apoptosis was generally present in spermatogonia cells (arrow head). (c) Widespread apoptosis in epididymal epithelium cells (arrow head) in the MTX group. (d) in the MTX + RES group, rare apoptotic cells (arrow head) and widespread normal epithelial cells (arrow) in epididymal epithelium (TUNEL analysis ×200).

TUNEL analysis results for the groups are given in Table 2. A low level of apoptosis in the spermatogonia was observed in the control and RES groups at analysis of testicular apoptosis. The percentage of TUNEL (+) apoptotic cells in the MTX group was significantly higher than that in the control and RES groups. These decreased significantly in the MTX + RES group compared to the MTX group. In the MTX group, apoptosis was particularly observed in the spermatogenic cells of the germinal epithelium and the germinal epithelial cells in the lumen (Figure 2(a)). In the MTX + RES group, it was seen more particularly in the spermatogonia and primary spermatocytes (Figure 2(b)). EAI was significantly high in the MTX group. It was also significantly lower in the MTX + RES group compared to the MTX group (Figures 2(c) and 2(d)).

## 4. Discussion

MTX is a cytotoxic chemotherapeutic drug widely used for malignancies and autoimmune diseases [20]. Testicular toxicity is an important side effect of MTX [5, 6]. Protection of germinal cells is important during the use of chemotherapeutics. In this study, our aim was to investigate whether there would be a change in the oxidative stress status of the MTX-administrated rat testis and whether use of RES can prevent these effects. 

Oxidative stress develops in association with an imbalance between reactive oxygen radicals (ROS) and the antioxidant reserve system. ROS are the product of normal cellular metabolism. Sperm is one type of cell that manufactures free oxygen radicals. With reactive oxygen radical production at low levels, sperm cell capacitation, acrosome reaction, and sperm binding to the zona pellucida take place [21]. Uncontrolled ROS production leads to sperm abnormalities and infertility. Sperm membrane is rich in polyunsaturated fatty acids. This leads to a rise in oxygen-induced lipid peroxidation [22]. Peroxidative damage is an important factor in sperm function impairment [23]. Sperm damage induced with MTX has been reported to be associated with oxidative stress. MDA is a lipid peroxidation level marker [5]. In this study, MTX caused increase in plasma and tissue MDA concentrations in the MTX group. This rise in MDA levels is in agreement with several previous studies demonstrating that MTX induces oxidative stress in tissues by increasing MDA levels [6, 20, 24, 25].

Antioxidant defense mechanisms in the testis are important in the protection of sperm against ROS. Spermatozoa combat ROS with such protective biomolecules as various antioxidants, vitamins, and glutathione [26]. SOD is one of the major antioxidant enzymes that protect the male reproductive organs against the harmful effects of ROS [27]. Hydrogen peroxide (H_2_O_2_)_,_ less effective than the superoxide group, is neutralized by being converted into products with a weaker effect, such as water and oxygen, by enzymes present in tissue, such as catalase and glutathione [28]. We investigated whether the antioxidant enzymes SOD and CAT have a protective effect that eliminates free radicals forming with MTX. SOD plays an important role in testicular development and spermatogenesis. Changes that take place in this enzyme may lead to impaired testicular functions and cessation of sperm development [26]. In this study, MTX caused a lowering of SOD and CAT activity in testicular tissue. We think that the low SOD and CAT enzyme levels may be associated with the rise in consumption and imbalance of resynthesis machinery.

Our histopathological findings support the biochemical results. The MTX group had a lower testis biopsy score than the control group. Widespread immature germinal cells were present in the seminiferous tubular lumen in the MTX group. This shows that spermatogenesis was not completed and was impaired [2, 3]. These alterations may be due to oxidative properties of MTX [5, 6]. 

A rise in ROS leads to cell damage and oxidative injury to DNA and proteins by increasing lipid peroxidation. Oxidative stress is an important mediator of apoptosis. Mitochondria play a significant role in the apoptotic process. Oxidative stress causes an impairment in mitochondria functions, cytochrome c release, and subsequent caspase activation. This concludes with apoptotic cell death [29]. Previous studies with anticancer drugs have shown that these drugs reduce germ cell numbers by increasing apoptosis in testicular germinal cells [5, 26]. In addition to its importance in normal testicular physiology, increased apoptosis also plays a significant role in the impairment of spermatogenesis [4, 30]. We used the TUNEL analysis technique to identify apoptotic DNA fragmentation. The TUNEL analysis results support those studies reporting that MTX damages DNA synthesis increases oxidative stress [5, 6] and leads to cell death [5]. The AI in the testicular germinal epithelial and epididymal epithelial cells was significantly higher in the MTX group. The administration of RES before MTX significantly lowered the AI in the seminiferous tubule and epididymis. Uguralp et al. [31] reported that RES reduced bilateral testicular MDA levels and germ cell apoptosis in the ipsilateral testis after testicular ischemia/reperfusion injury. Paiva et al. [32] reported that ROS triggers apoptotic cell death. We therefore concluded that RES prevents the formation of ROS and thus causes a decrease in AI. 

The results of our study show that MTX leads to harmful side effects in testicular tissue. We think that these effects are associated with its preventing DNA synthesis and causing oxidative stress by leading to excessive production of ROS [5].

The administration of RES before MTX partly or entirely neutralized these effects in this study. Plasma and tissue MDA concentrations were significantly lower and SOD enzyme activity significantly higher in the MTX + RES group. Previous studies have reported that RES reduces oxidative stress by lowering MDA concentrations in tissue [9, 33]. Seminal plasma and sperm are protected against cellular damage by antioxidant enzymes, such as glutathione peroxidase (GP-x), SOD, and CAT and by antioxidant substances, such as vitamins C and E [34]. Studies of antioxidant enzymes have reported that RES is a free radical scavenger and enzyme regulator and therefore protects against tissue damage caused by oxidative stress. Additionally, one study showed that RES can serve like the antioxidant enzymes SOD1 and GPx1 [35]. Histologically, our study showed that RES treatment significantly protected testicular seminiferous tubules against MTX toxicity. The protective effect of RES treatment may be due to its protection of cellular membranes against oxidative damage [5, 31].

## 5. Conclusion

Our study shows that oxidative stress is important in MTX-induced testicular damage. The administration of RES reduced oxidative stress and apoptotic cell death and protected spermatogenesis in MTX-induced oxidative testicular damage. We think that this protective effect of RES may be due to its antioxidant property. However, this study needs to be supported with other experimental and clinical research. 

## Figures and Tables

**Table 1:** Oxidant and antioxidant biochemical parameters in all groups.

Parameters	Control	RES	MTX	MTX + RES
Plasma MDA (nmol/L)	131.77 ± 20.14	139.58 ± 11.45	231.30 ± 34.15^a^	172.11 ± 13.76^a,b^
Tissue MDA (nmol/g)	175.57 ± 14.31	178.15 ± 13.92	229.02 ± 43.53^a^	182.44 ± 9.25^a,b^
SOD (U/g tissue)	428.77 ± 23.49	424.70 ± 35.56	348.73 ± 29.51^a^	462.45 ± 24.41^b^
CAT (k/g)	0.87 ± 0.09	0.79 ± 0.05	0.5 ± 0.09^a^	0.65 ± 0.03^a^

^a^ *P* < 0.05, when compared to the control and RES groups.

^b^ *P* < 0.05, when compared to the MTX group.

**Table 2:** Testis histopathologic damage scores and apoptotic index in all groups.

Parameters	Control	RES	MTX	MTX + RES
JTBS	9.7 ± 0.2	9.66 ± 0.2	6.55 ± 0.22^a^	8.93 ± 0.39^a,b^
TAI (%)	1.94 ± 0.5	2.28 ± 0.3	28.66 ± 2.8^a^	15.66 ± 2.06^a,b^
EAI (%)	7.56 ± 0.51	6.95 ± 0.8	41.8 ± 3.13^a^	22.73 ± 2.56^a,b^

^a^ *P* < 0.05, when compared to the control and RES groups.

^b^ *P* < 0.05, when compared to the MTX group.
